# Quality matters in strengthening global laboratory medicine

**DOI:** 10.4102/ajlm.v3i2.239

**Published:** 2014-11-03

**Authors:** John N. Nkengasong, Deborah Birx

**Affiliations:** 1 Division of Global HIV/AIDS, Center for Global Health, US Centers for Disease Control and Prevention, Atlanta, United States

## Introduction

Around 300 BC, during the time of the ancient Greek physician, Hippocrates, the first documented examination of human bodily fluids was conducted. This gave birth to laboratory medicine, which is the use of laboratory tests to guide clinical investigations.^1^ Ever since, as a result of its multi-faceted nature, ensuring the quality of testing in laboratory medicine has remained a challenge, but is an evolving practice in many countries. In the developed world, laboratory medicine has been transformative and, in most cases, is the science behind clinical care and disease surveillance. Central to the practice of laboratory medicine in the developed world is the recognition of the importance of quality assurance (QA). As such, there is regular review of existing policies in order to ensure the continuous improvement of quality systems. For instance, in the United States, reports by the Institute of Medicine, *To err is human: building a safer health system* (1999),^2^ and The Committee on Quality of Health Care in America, *Crossing the quality chasm: a new health system for the 21st century* (2001),^3^ helped refocus attention on the need to minimise medical errors and improve quality.

**FIGURE 1:**
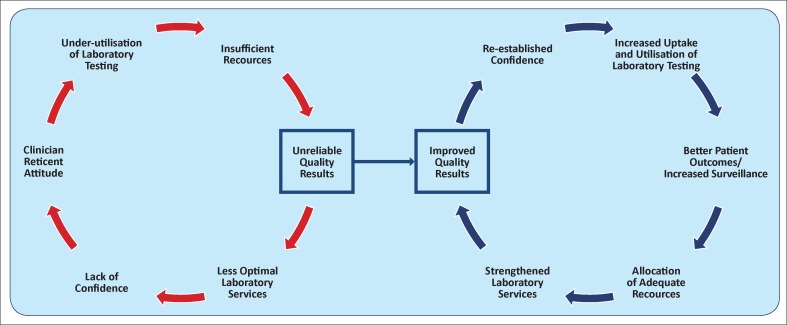
Quality matters: A catalyst to a tipping point for strengthening laboratory medicine?

In developing countries, QA in laboratory medicine has been severely neglected and has become a serious impediment to effective healthcare delivery and disease surveillance.^4,5,6^ In fact, a vicious cycle has been established whereby most physicians in developing countries rely on history-taking and physical examination for patient management, since they have little confidence in laboratory test results, even if laboratory facilities exist.^5^ As such, inadequate resources are allocated to laboratory services, which in turn results in less-than-optimal quality-assured results, further leading to the neglect of laboratory systems (Figure 1). Nonetheless, many countries are currently making great strides in implementing quality management systems (QMS), which is leading to laboratory accreditation to international standards.^7,8^ The importance of quality in laboratory medicine cannot be overstated: it adds significant value to patient outcomes and management;^9^ reduces wastage, minimises sample rejection and enhances client satisfaction;^10^ prevents unneeded diagnostic testing; improves turnaround times for accurate diagnosis; and reduces the use of inappropriate treatment. Because laboratory errors occur at a rate of 32% – 75% in the pre-analytic phase, 13% – 32% in the analytic phase and 9% – 31% in the post-analytic phase,^11,12^ it is vital that assuring the quality of laboratory medicine be considered a continuum of a total testing process of all analytical phases. Errors that occur in the pre-analytical phase in the spectrum of laboratory testing can have a direct effect on patient outcomes in the post-analytical stage.

### Squaring the circle

Because of the acute challenges of implementing QA in laboratory medicine, healthcare providers in developing countries have unwisely neglected the important role of laboratory diagnosis in patient care. As a result, achieving the International Organization for Standardization (ISO) 15189 requirements for clinical laboratory has become a lofty aspiration. Understandably, but unfortunately, these countries seem to find themselves at an impasse of practising laboratory medicine in the 21st century by squaring the circle: relying on inadequate quality-assured test results or empiricism for patient management, resulting in disproportionate administration of antibiotics and high cost to patients.^5^ In the last decade, there has been a massive focus on global health, with funding reaching an unprecedented US $28.2 billion in 2010.^13^ The surge in funding has resulted in the recognition that strengthening health systems, including laboratory services, is critical to healthcare delivery. Thus, an unparalleled opportunity has presented itself to strengthen quality-assured laboratory medicine, using innovative approaches to addressing old, neglected challenges.

### Time to stop doing more of the same

Increased funding for global health has placed a sense of urgency on stakeholders to act now and collectively, but in a different manner.^14^ Previous approaches that attempted to strengthen QMS did not use a holistic approach, focusing rather on individual activities: QA, proficiency testing and theoretical concepts. This approach has shown severe limitations with regard to advancing quality-assured laboratory medicine in developing countries. To continue with such strategies would be analogous to pounding square pegs into round holes. Rather, novel holistic approaches that place emphasis on task-based and results-driven quality improvement projects are needed urgently. To achieve these goals, laboratory medicine in developing countries must innovate and create performance-enablers that would both incentivise and energise the field, thereby facilitating adoption. Successful performance-enablers must focus on four chief aspects: implementation, measurement, reward and improvement. In order to ensure a sustainable culture of the practice of quality-assured laboratory medicine, countries need to embrace innovative QMS programmes, which require commitment to the process of continuous quality improvement of laboratory medicine. Some countries have made remarkable progress by using innovative approaches to implementing laboratory accreditation. For example, between 1961 and 1998, South Korea endorsed the Laboratory Accreditation Program (LAP) of the College of American Pathologists (CAP); however, during that time only 11 laboratories were accredited.^15^ Because of the challenges in implementation of LAP, in 1999, South Korea modified the CAP-LAP into a step-wise laboratory accreditation process known as the Korea Laboratory Accreditation Process (KLAP). In KLAP, laboratories with a score of > 90% received a two-year certificate; laboratories with scores between 60% and 89% received a one-year certificate; and those with a score of < 60% failed the certification. As a result of KLAP, 227 laboratories were certified between 1999 and 2006 and many laboratories were enrolled in the programme across the country. In 2001, Thailand established a step-wise national accreditation programme as a local alternative for improvement of laboratory quality. The accreditation programme was established with standards based on ISO 15189 and, from 2003 to 2009, 724 (50.6%) of 1432 laboratories in Thailand were assessed. Of these, 197 (27.2%) were accredited, primarily in the government sector.^16^ The programme has thus far been affordable, feasible, scalable, sustainable and effective.^16^

Over the past five years, through global partnership, innovative performance-enablers have been developed to guide the implementation of a sustainable QMS leading to accreditation in developing countries: (1) the Stepwise Laboratory Quality Improvement Process Towards Accreditation (SLIPTA);^17^ (2) the Caribbean Laboratory Quality Management System – Stepwise Improvement Process (LQMS-SIP) towards Accreditation;^18^ and (3) the task-based Strengthening Laboratory Management Toward Accreditation (SLMTA) programme.^19^

### SLIPTA and LQMS-SIP, the companions of SLMTA

Both SLIPTA and LQMS-SIP are innovative performance-enabling tools and companions of SLMTA. These tools were designed to motivate the implementation of QMS with measurable delivery through SLMTA.

SLIPTA is a framework endorsed by the World Health Organization Regional Office for Africa (WHO AFRO) and jointly implemented by the African Society for Laboratory Medicine (ASLM) for improving the quality of public health laboratories in African countries to achieve accreditation to the ISO 15189 standard. The WHO AFRO SLIPTA Checklist is based on ISO 15189/17025, with 111 items and a possible 258 points, which are further divided into five star levels: one to five.^17^

LQMS-SIP has been endorsed in the Caribbean region and consists of a three-tiered system: the first tier represents the minimum requirements that correspond to mandatory criteria required for the granting of a licence based on legislation enacted by the Ministries of Health. The next two tiers are quality-improvement levels representing achievements in meeting specific requirements of a QMS. The Caribbean Regional Organization for Standards and Quality hosts the LQMS-SIP secretariat and works directly with countries and other laboratory stakeholders to coordinate the rollout and implementation of the regulatory activities and the recognition process. The Caribbean Public Health Agency (CARPHA) helps with the coordination of the Caribbean Public Health Laboratory Network and participates in the development of quality standards.

## Evident evidence and the up-shot

SLMTA is grounded in its ability to identify deficiencies in a laboratory, improve them and measure the outcomes. After completing a full SLMTA round, which typically lasts for up to 18 months, changes are evident and outcomes are visible. Laboratories experience a net progression from one to five stars on the SLIPTA scoring checklist upon completion of the SLMTA programme. More significant is the enduring impact on personnel who have undergone SLMTA training, achieving positive changes in attitude toward the culture of quality, as well as recognition of quality-assured laboratory medicine. In this special issue of the *African Journal of Laboratory Medicine*, several countries have shared remarkable evidence regarding how SLMTA has been transformative in their laboratories^7,8^ and is beginning to stimulate changes in hospital management.^20^ Since 2009, when SLMTA was launched in Kigali, Rwanda, it has expanded exponentially. As of the end of 2013 it has been implemented in 47 countries in Africa, the Caribbean, Latin America and Southeast Asia. With the introduction of SLMTA, the prospects of implementing sustainable quality-assured laboratory medicine seem to be a reality in developing countries. In total, the 302 laboratories that have completed the SLMTA programme conduct approximately 43.5 million diagnostic tests annually. Based on baseline audit scores, laboratories that had at least one quality star prior to SLMTA participation conducted only one out of every six tests. This number quadrupled to two out of three after SLMTA training. These gains have also proven to be sustainable; of the 92 laboratories that have conducted surveillance audits at five to 28 months after SLMTA completion, 62% showed a further increase in their score from the exit audit, with more than half increasing their score by more than 10 additional percentage points.^8^ At present, countries that have implemented SLMTA are caught between a state of cautious optimism and open-minded concern about the rollout and sustainability of the programme.

### SLMTA, nearing the tipping point?

In order to ensure sustainability, it is urgent to identify system drivers that will enable the country to reach a tipping point; these include expanded coverage and demonstration of the impact on patient care. Such a tipping point, defined in this context as the number of laboratories that will constitute a critical mass for the demand of the programme to become a nation-wide requirement, once attained, will begin to increase confidence in quality-assured laboratory medicine for evidence-based patient management. This may lead to an increased uptake and use of laboratory test results, encouraging greater investment of resources in laboratory services and, ultimately, breaking the vicious cycle of the neglected laboratory systems in developing countries (Figure 1). Potential drivers that could facilitate a tipping point for SLMTA include incorporating SLMTA into a pre-service curriculum for schools of medical laboratory sciences; strengthening the clinical-laboratory interface; developing country-specific national strategic plans for rolling out SLMTA and other QMS tools; accelerating the process by engaging ASLM or similar organisations to audit and reward laboratories that have undergone the SLMTA process; incorporating basic laboratory information systems as part of QMS; designing SLMTA-like training tools for hospital and clinic certification and accreditations; and, lastly, encouraging donors and funders to prioritise QA and continuous quality improvement as a core component of laboratory health system strengthening.

### Conclusion

SLMTA eliminates redundant procedures by reorganising the laboratory set-up so that personnel spend less time handling and processing specimens. SLMTA is not a destination; rather, it is a journey that relies on ongoing cooperation at all levels, including senior management, laboratory staff and end users.

In the 21st century, as the economies of developing countries continue to grow, many individuals will begin to seek affordable quality-assured healthcare; as such, there is bound to be increasingly consumer-oriented healthcare that holds physicians and laboratory workers more accountable for errors. Because of improved quality-assured laboratory medicine, healthcare providers will broaden their practice from using laboratory tests to confirm clinical diagnoses to using tests to detect clinically unapparent diseases, as well as to support outbreak surveillance responses, such as the recent outbreak of Ebola virus in West Africa. In order to meet this demand, both SLMTA and SLIPTA will need to be rolled out in developing countries so as to stimulate healthcare service providers to focus on a systematic work flow for quality services rendered to patients, resulting in increased efficiency and quality whilst lowering waste and cost and improving safety. Critically, a patient-centered continuous quality improvement approach will become indispensable. Hospital accreditation, as in Thailand, will most likely drive the need for laboratory quality, as active involvement of managers will create collective organisational commitment of quality improvement and a focus on patient-oriented thinking.
